# A machine learning-based prediction of diabetic retinopathy using the Korea national health and nutrition examination survey (2008–2012, 2017–2021)

**DOI:** 10.3389/fmed.2025.1542860

**Published:** 2025-05-30

**Authors:** Min Seok Kim, Young Wook Choi, Borghare Shubham Prakash, Youngju Lee, Soo Lim, Se Joon Woo

**Affiliations:** ^1^Department of Ophthalmology, Seoul National University College of Medicine, Seoul National University Bundang Hospital, Seongnam-si, Republic of Korea; ^2^RetiMark R&D Center, Seoul, Republic of Korea; ^3^Department of Internal Medicine, Seoul National University College of Medicine and Seoul National University Bundang Hospital, Seongnam-si, Republic of Korea

**Keywords:** diabetic retinopathy, machine learning, random forest algorithms, Korea, prediction

## Abstract

**Background:**

Machine learning technology that uses available clinical data to predict diabetic retinopathy (DR) can be highly valuable in medical settings where fundus cameras are not accessible.

**Objective:**

This study aimed to develop and compare machine learning algorithms for predicting DR without fundus image.

**Methods:**

We used data from Korea National Health and Nutrition Examination Survey (2008–2012 and 2017–2021) and enrolled individuals aged ≥ 20 years with diabetes who received fundus examination. Predictive models for DR were developed using logistic regression and three machine learning algorithms: extreme gradient boosting, decision tree, and random forest. Model performance was evaluated using area under the receiver operating characteristic curve (AUC) and accuracy for the diagnosis of DR, and feature importance was determined using Shapley Additive Explanations (SHAP).

**Results:**

Among the 3,026 diabetic participants (male, 50.7%; mean age, 63.7 ± 10.5 years), 671 (22.2%) had DR. The random forest model, using 16 variables, achieved the highest AUC of 0.748 (95% confidence interval, 0.705–0.790) with a sensitivity 0.669, specificity of 0.729 and an accuracy of 0.715. As interpreted by SHAP, HbA1c, fasting glucose levels, duration of diabetes, and body mass index were identified as common key determinants influencing the model’s outcomes.

**Conclusion:**

The DR prediction models using machine learning techniques demonstrated reliable performance even without fundus imaging, with the random forest model showing particularly strong results. These models could assist in managing DR by identifying high-risk patients, enabling timely ophthalmic referrals.

## Introduction

Diabetes mellitus is one of the major chronic diseases, and its prevalence is rapidly increasing worldwide. One of the severe complications of diabetes is diabetic retinopathy (DR), a leading cause of blindness among working-age adults worldwide (1). Globally, it is estimated that approximately one-third of patients with diabetes have some form of DR, with the prevalence ranging from 35 to 40% (2). In 2019, it was reported that around 146 million people were affected by DR, and this number is projected to increase to 191 million by 2030 (3). Early detection and intervention are crucial in preventing severe vision loss and improving the quality of life for diabetic patients, as DR often presents without symptoms even in late stages (4). However, non-adherence to DR examination is common among diabetic patients globally, and thus, a better way to screen high-risk patients for DR is required (5–8). Recently, the evolution of machine learning technology has sparked significant interest and gained popularity, particularly for enhancing clinical decision-making (9). Machine learning techniques enable clinicians to leverage complex datasets to predict outcomes such as disease progression, treatment response, and patient outcomes with high accuracy and efficiency (10, 11). In medical settings without access to a fundus camera, machine learning technology that utilize available clinical data to predict DR can be highly beneficial.

This study aims to develop a machine learning model to predict the risk of DR in diabetic patients using clinical variables from Korea National Health and Nutrition Examination Survey (KNHANES) data to provide an efficient and accurate diagnostic support system for DR in clinical settings.

## Materials and methods

### Study design and participants

**FIGURE 1:**
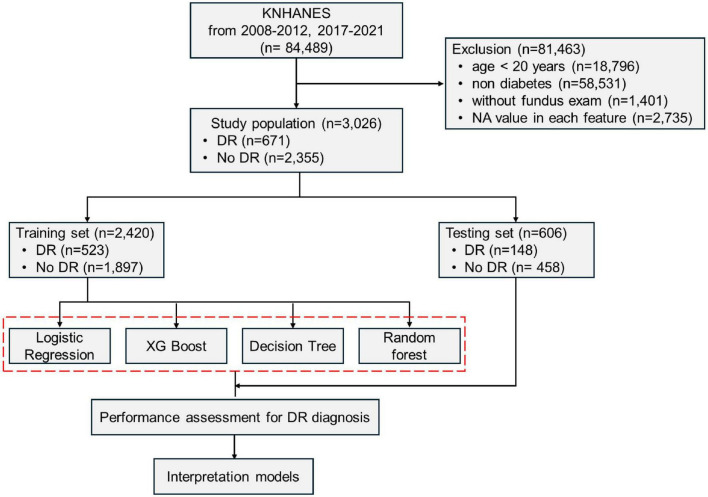
Flow diagram for the training and testing sets of the logistic regression and the three machine learning systems. KNHANES, Korea National Health and Nutrition Examination Survey; DR, diabetic retinopathy.

This study was conducted using the KNHANES data (2008–2012 and 2017–2021) (12). Based on self-reported questionnaire-based information, the participants who answered, “yes” to the question “Have you ever been diagnosed with diabetes by a doctor before?” were classified as diabetic patients. Participants were selected based on the following criteria: age over 20, diagnosed with diabetes, having fundus examination results, and no missing data in the variables used for analysis. The final study sample included 3,026 participants. To develop and evaluate a predictive diagnostic model for DR, the sample was split into a training set (2,420 participants) and a test set (606 participants) in an 8:2 ratio (Figure 1). DR was identified based on the presence of any characteristic lesions defined by the Early Treatment Diabetic Retinopathy Study severity scale using a non-mydriatic fundus camera. Grading was performed by experienced retinal specialists, with DR diagnosed in the presence of microaneurysms, hemorrhages, hard exudates, cotton wool spots, intraretinal microvascular abnormalities, venous beading, or retinal neovascularization (12). DR grading was not additionally assessed in all years, whereas the presence or absence of DR was determined.

### Model development and performance evaluation

We developed prediction models using logistic regression and three machine learning algorithms: extreme gradient boosting (XGB), Decision Tree (DT), and random forest (RF). These algorithms employ various approaches to map predictor values to probabilities between zero and one. The evaluation of predicted performance was carried out using the area under the receiver operating characteristic curve (AUC) along with 95% confidence intervals (CI) for each prediction model (13). A comparison was made between the performance of the logistic regression and the machine learning models. Additionally, performance was measured using sensitivity, specificity, positive predictive value, and negative predictive value, all of which were determined based on a single cut-off value designed to maximize the Youden index (calculated as sensitivity + specificity - 1). Model calibration was assessed by comparing the observed event probabilities with the predicted ones.

To enhance the random forest model’s performance, we applied hyperparameter tuning using RandomizedSearchCV, a method that efficiently samples a wide range of hyperparameter combinations to identify the optimal settings. This approach is particularly advantageous when dealing with large datasets or numerous hyperparameters, as it reduces computational cost compared to exhaustive methods like GridSearchCV.

For the random forest model, we tuned hyperparameters including the number of trees (*n_estimators: [100, 150, 173, 200, 250, 600]*), maximum tree depth (*max_depth: [10, 15, 20, None]*), minimum samples to split a node (min_samples_split: [2, 4, 6, 10]), minimum samples at a leaf (*min_samples_leaf: [1, 2, 4, 6]*), features considered for splitting *(max_features: [’auto’, ‘sqrt’, ‘log2’]*), sampling method (*bootstrap: [True, False]*), and splitting criterion (*criterion: [’gini’, ‘entropy’]*). The optimal parameters identified were *n_estimators* = *173, max_depth* = *None, min_samples_split* = *6, min_samples_leaf* = *4, max_features* = *’sqrt’, bootstrap* = *True, and criterion* = *’entropy’*.

For the Logistic Regression model, hyperparameter optimization focused on the regularization strength (*C: np.logspace*(−4, 4, 20)), penalty type (*penalty: [’l1’, ‘l2’] for Lasso and Ridge regularization*), and solver algorithm (*solver: [’liblinear’, ‘saga’]*). These parameters helped improve model generalization and prevent overfitting.

For the Decision Tree model, the grid search evaluated the split criterion (*criterion: [’gini’, ‘entropy’]*), split strategy (*splitter: [’best’, ‘random’]*), maximum depth (*max_depth: [None, 5, 10, 15, 20]*), minimum samples for node splitting (*min_samples_split: [2, 5, 10]*), minimum samples at leaf nodes (*min_samples_leaf: [1, 2, 4]*), and features for splitting (*max_features: [’sqrt’, ‘log2’]*). These settings enhanced model complexity control and interpretability.

For the XGBoost model, tuning focused on the learning rate (*learning_rate: [0.1, 0.01, 0.001]*), tree depth (*max_depth: [3, 5, 7]*), minimum child weight (*min_child_weight: [1, 3, 5]*), training data subsampling (*subsample: [0.8, 0.9, 1.0]*), features used per tree (*colsample_bytree: [0.8, 0.9, 1.0]*), and boosting rounds (*n_estimators: [100, 200, 300]*). The grid search approach optimized the model’s ability to learn complex patterns while maintaining generalization.

### Feature selection techniques

Recursive Feature Elimination with Cross-Validation (RFECV) was used with 5-fold cross-validation. RFECV is a robust method for feature selection that iteratively removes the least important features based on model performance until the optimal subset is found. By using 5-fold cross-validation, the model’s performance is validated across different subsets of data, reducing the risk of overfitting. This approach ensures that only the most relevant features contribute to the predictive model, enhancing both accuracy and interpretability.

### Feature Analysis using SHapley Additive exPlanations (SHAP)

SHAP interprets machine learning models by showing feature importance and their impact on predictions. It uses values from game theory to quantify each feature’s contribution to the model’s output. This helps data scientists and stakeholders understand and trust the model, enhancing transparency and enabling model refinement (14).

We used a broad spectrum of patient characteristics and health indicators as variables in our analysis, including age, sex, duration and treatment of diabetes mellitus, smoking status, comorbidities, waist circumference, systolic blood pressure, diastolic blood pressure, body mass index (BMI), and laboratory results.

### Statistical analysis

The continuous variables were analyzed using the independent *t*-test, and the categorical variables were analyzed and compared by the Chi-squared or Fisher’s exact tests. All statistical analysis was conducted with Python (version 3.12.2). The statistical significance criterion was set to be two-sided, and *p*-values < 0.05 were considered statistically significant.

### Ethical adherence

This study was reviewed and approved by the Institutional Review Board (IRB) of Seoul National University Bundang Hospital (IRB No: X-2212-796-901). Informed consent was waived by the IRB due to the anonymized data and retrospective design of the study. The study followed the principles of the Declaration of Helsinki.

## Results

A total of 3,026 diabetic participants aged ≥ 20 years were included in the analysis. All the participants had fundus examination results, with a prevalence of DR of 22.2% (*n* = 671). The baseline characteristics of the training and test datasets are shown in Table 1.

**TABLE 1:** Comparison of training and test data between DR and no DR.

Feature	Total data set (*n* = 3026)			Train data set (*n* = 2420)			Test data set (*n* = 606)		
	**DR** **(*n* = 671)**	**No DR** **(*n* = 2355)**	***P*-value**	**DR** **(*n* = 523)**	**No DR** **(*n* = 1897)**	***P*-value**	**DR** **(*n* = 148)**	**No DR** **(*n* = 458)**	***P*-value**
Male, sex, no. (%)	347 (51.7)	1188 (50.5)	0.583	275 (52.6)	956 (50.4)	0.373	72 (48.7)	232 (50.7)	0.673
Age, years, no. (%)	62.9 (10.3)	63.9 (10.5)	0.033	63.0 (10.4)	64.0 (10.4)	0.039	62.7 (10.0)	63.3 (10.9)	0.559
< 40	10 (1.5)	34 (1.4)	0.847	7 (1.3)	25 (1.3)	1.000	3 (2.0)	9 (1.9)	0.939
40∼50	62 (9.2)	215 (9.1)	0.937	51 (9.8)	163 (8.6)	0.393	11 (7.4)	52 (11.4)	0.167
50∼60	169 (25.2)	516 (21.9)	0.072	130 (24.9)	417 (22.0)	0.161	39 (26.4)	99 (21.6)	0.227
60∼70	237 (35.3)	779 (33.1)	0.287	178 (34.0)	626 (33.0)	0.667	59 (39.9)	153 (33.4)	0.150
> 70	193 (28.8)	811 (34.4)	0.007	157 (30.0)	666 (35.1)	0.029	36 (24.3)	145 (31.7)	0.088
DM duration, years	12.4 (9.3)	8.0 (7.7)	<0.001	12.0 (8.4)	8.1 (7.8)	<0.001	12.1 (8.7)	7.8 (7.3)	<0.001
Smoking, current, No. (%)	128 (19.1)	398 (16.9)	0.185	99 (18.9)	315 (16.6)	0.216	29 (19.6)	83 (18.1)	0.683
**Co-morbidity, No. (%)**									
Hypertension	373 (55.6)	1455 (61.8)	0.004	288 (55.1)	1175 (61.9)	0.005	85 (57.4)	280 (61.1)	0.424
Hyperlipidemia	302 (45.0)	1135 (48.2)	0.143	239 (45.7)	900 (47.4)	0.491	63 (42.6)	235 (51.3)	0.066
Stroke	48 (7.2)	121 (5.1)	0.036	34 (6.5)	95 (5.0)	0.176	14 (9.5)	26 (5.7)	0.107
Myocardial infarction	24 (3.6)	70 (3.0)	0.431	18 (3.4)	61 (3.2)	0.819	6 (4.1)	9 (2.0)	0.156
Diabetic nephropathy	11 (1.6)	18 (0.76)	0.047	10 (1.9)	12 (0.63)	< 0.001	1 (0.68)	6 (1.3)	0.539
**Other eye disease, No (%)**									
Glaucoma	100 (14.9)	355 (15.1)	0.898	84 (16.1)	293 (15.5)	0.738	16 (10.8)	62 (13.5)	0.394
AMD	98 (14.6)	368 (15.6)	0.527	77 (14.7)	293 (15.5)	0.653	21 (14.2)	75 (16.4)	0.525
WC, cm	88.0 (8.8)	88.3 (9.4)	0.463	88.3 (8.9)	88.4 (9.6)	0.814	87.2 (8.4)	88.1 (8.8)	0.276
SBP, mmHg	127.8 (17.2)	125.9 (16.3)	0.011	127.8 (17.4)	126.2 (16.5)	0.050	127.6 (16.5)	124.7 (15.5)	0.059
DBP, mmHg	73.8 (10.3)	74.6 (9.9)	0.059	73.6 (10.6)	74.6 (10.0)	0.064	74.5 (9.1)	75.0 (9.3)	0.570
FBG, g/dL	153.5 (49.9)	133.0 (36.1)	<0.001	150.8 (46.9)	132.7 (35.2)	<0.001	163.1 (58.7)	134.1 (39.5)	<0.001
HbA1c,%	7.9 (1.6)	7.1 (1.2)	<0.001	7.8(1.5)	7.1 (1.2)	<0.001	8.1(1.7)	7.1 (1.2)	<0.001
BMI, kg/m^2^	24.7 (3.3)	25.1 (3.4)	0.008	24.7 (3.3)	25.1 (3.5)	0.032	24.6 (3.3)	25.1 (3.1)	0.093
TC, mg/dL	171.3 (41.8)	171.3 (38.4)	0.976	171.5 (41.0)	171.6 (38.0)	0.958	170.6 (44.9)	170.3 (39.8)	0.943
TG, mg/dL	164.7 (125.6)	154.5 (110.2)	0.056	163.1 (118.1)	153.5 (105.6)	0.093	170.5 (149.4)	158.6 (127.4)	0.386
HDL-c, md/dL	45.0 (11.2)	45.9 (11.2)	0.083	44.9 (10.5)	45.9 (11.2)	0.083	45.4 (13.5)	45.9 (11.6)	0.673
**DM treatment**									
Insulin	111 (16.5)	102 (4.3)	<0.001	87 (16.6)	79 (4.2)	<0.001	24 (16.2)	23 (5.0)	<0.001
OHA	623 (92.9)	2097 (89.1)	0.004	490 (93.7)	1694 (89.3)	0.003	133 (89.9)	403 (88.0)	0.530
AST, U/L	24.5 (14.9)	25.9 (12.1)	0.024	24.6 (15.2)	25.7 (11.5)	0.118	24.2 (13.9)	26.8 (14.0)	0.051
ALT, U/L	25.1 (23.0)	26.1 (17.5)	0.281	25.5 (24.6)	25.8 (16.7)	0.805	23.5 (16.1)	27.4 (20.6)	0.019
Hemoglobin, g/dL	13.7 (1.6)	13.9 (1.6)	0.023	13.8 (1.6)	13.9 (1.6)	0.040	13.7 (1.7)	13.9 (1.6)	0.383
BUN, mg/dL	17.6 (6.4)	16.8 (5.5)	0.007	17.6 (6.3)	16.8 (5.5)	0.005	17.3 (6.9)	17.0 (5.5)	0.616
Serum creatinine (mg/dL)	0.90 (0.53)	0.87 (0.27)	0.051	0.90 (0.39)	0.87 (0.27)	0.104	0.95 (0.85)	0.87 (0.28)	0.249
WBC, 10^6^/uL	6.67 (1.7)	6.60 (1.8)	0.368	6.62 (1.7)	6.60 (1.8)	0.741	6.8 (1.8)	6.6 (1.9)	0.206
RBC, 10^6^/uL	4.46 (0.5)	4.52 (0.5)	0.014	4.47 (0.5)	4.52 (0.5)	0.042	4.4(0.5)	4.5 (0.5)	0.173

Values are presented as mean and standard error or number and percentage. DR, diabetic retinopathy; BMI, body mass index; WC, waist circumference; SBP, systolic blood pressure; DBP, diastolic blood pressure; HbA1c, glycosylated hemoglobin; FBG, fasting blood glucose; TC, total cholesterol; HDL-C, high-density lipoprotein cholesterol; TG, triglyceride; OHA, oral hypoglycemic agents; BUN, blood urea nitrogen ratio; WBC, white blood cell; RBC, red blood cell; AMD, age-related macular degeneration.

**FIGURE 2:**
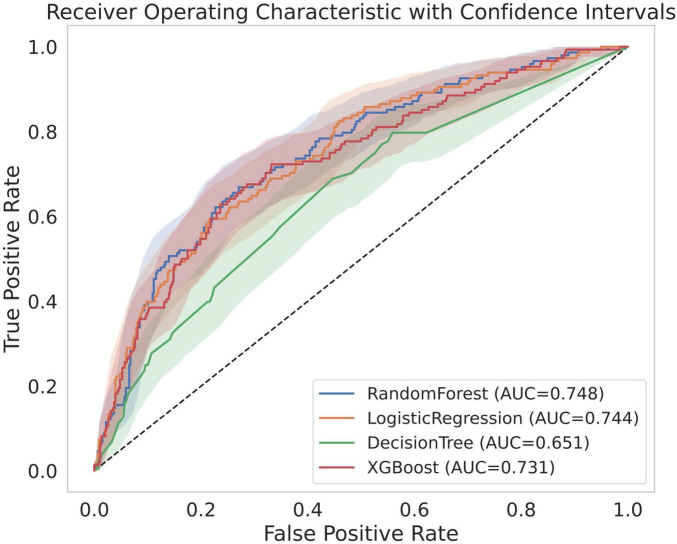
Receiver operating characteristic curves of models. AUC, area under the receiver operating characteristic curve.

The predictive performances and test characteristics of the models derived using logistic regression analysis and machine learning models are summarized in Table 2. In the validation using the test dataset, RF demonstrated the highest predictive performance achieving an AUC of 0.748 (95% CI, 0.705–0.790) with a sensitivity 0.669, specificity of 0.729 and an accuracy of 0.715. The AUC was 0.744 (95% CI, 0.701–0.788) in the logistic regression model, 0.731 (95% CI, 0.684–0.776) in the XGB model, and 0.651 (95% CI, 0.597–0.702) in the DT model (Figure 2).

**TABLE 2:** Discrimination and test characteristics of diabetic retinopathy prediction models.

Algorithm	AUC (95% CI)	Sensitivity (95% CI)	Specificity (95% CI)	Accuracy (95% CI)	PPV (95% CI)	NPV (95% CI)	Optimal threshold
Logistic regression	0.744 (0.701, 0.788)	0.595 (0.556, 0.634)	0.784 (0.746, 0.821)	0.738 (0.702, 0.772)	0.471 (0.430, 0.510)	0.857 (0.823, 0.890)	0.257
Extreme gradient boosting	0.731 (0.684, 0.776)	0.622 (0.583, 0.660)	0.764 (0.726, 0.803)	0.729 (0.693, 0.764)	0.460 (0.420, 0.499)	0.862 (0.829, 0.896)	0.235
Decision tree	0.651 (0.597, 0.702)	0.662 (0.624, 0.699)	0.576 (0.531, 0.621)	0.597 (0.559, 0.637)	0.336 (0.299, 0.373)	0.841 (0.800, 0.881)	0.185
Random forest	0.748 (0.705, 0.790)	0.669 (0.631, 0.706)	0.729 (0.689, 0.769)	0.715 (0.679, 0.750)	0.444 (0.404, 0.483)	0.872 (0.839, 0.906)	0.247

AUC, area under the receiver operating characteristic curve; NPV, negative predictive value; PPV, positive predictive value. In the validation using the test dataset, random forest model demonstrated the highest predictive performance.

**FIGURE 3:**
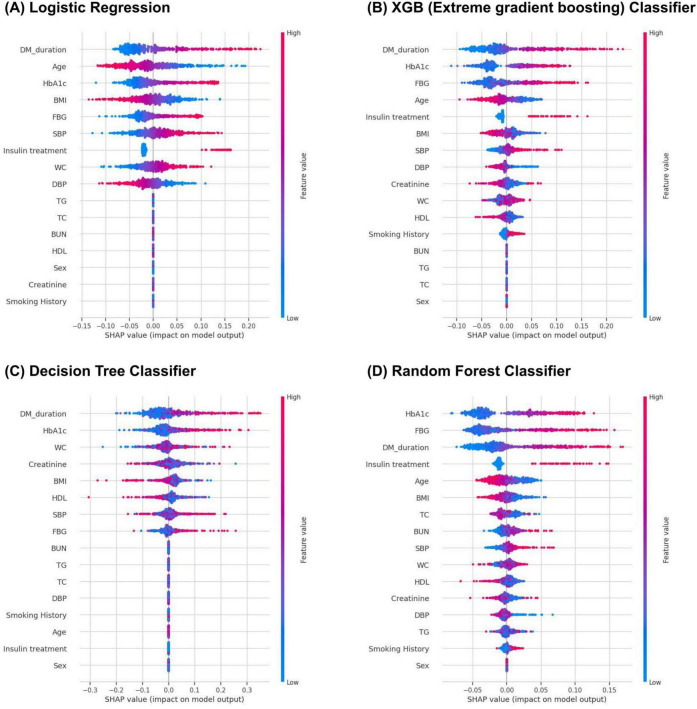
Feature importance score of logistic regression **(A)**, XGB **(B)**, decision tree **(C)**, and random forest **(D)** models.

As depicted in Figure 3 and Table 3, key features for prediction across all models were HbA1c levels, fasting blood glucose levels, duration of diabetes mellitus, and BMI among the top eight variables in each model. HbA1c (SHAP value, 0.044; effects, 0.183) was the most influential factor in the RF model outcomes, followed by fasting glucose levels (SHAP value, 0.043; effects, 0.180), duration of diabetes mellitus (SHAP value, 0.040; effects, 0.168), insulin usage (SHAP value, 0.018; effects, 0.074), age (SHAP value, 0.016; effects, 0.068), and other factors.

**TABLE 3:** The Shapley Additive Explanations (SHAP) values of the top eight variables of corporate risk-taking.

Algorithm	Feature	SHAP value	Effects
Logistic regression	DM duration	0.052	0.164
	Age	0.046	0.144
	HbA1c	0.041	0.128
	BMI	0.033	0.104
	FBG	0.032	0.101
	SBP	0.031	0.099
	Insulin usage	0.029	0.093
	WC	0.027	0.086
Extreme gradient boosting	DM duration	0.049	0.192
	HbA1c	0.043	0.168
	FBG	0.041	0.158
	Age	0.025	0.096
	Insulin usage	0.017	0.066
	BMI	0.017	0.064
	SBP	0.014	0.056
	DBP	0.012	0.048
Decision tree	DM duration	0.064	0.221
	HbA1c	0.050	0.172
	WC	0.034	0.118
	Creatinine	0.032	0.110
	BMI	0.031	0.106
	HDL	0.029	0.101
	SBP	0.028	0.096
	FBG	0.022	0.078
Random forest	HbA1c	0.044	0.183
	FBG	0.043	0.180
	DM duration	0.040	0.168
	Insulin usage	0.018	0.074
	Age	0.016	0.068
	BMI	0.011	0.047
	TC	0.010	0.041
	BUN	0.009	0.037

BMI, body mass index; WC, waist circumference; SBP, systolic blood pressure; DBP, diastolic blood pressure; HbA1c, glycosylated hemoglobin; FBG, fasting blood glucose; TC, total cholesterol; DM, diabetes mellitus; BUN, blood urea nitrogen.

## Discussion

This study aimed to develop an optimized machine learning model to predict the diagnosis of DR among adults with diabetes using the KNHANES data, a nationwide survey database representative of the South Korean population.

In this study, we developed various models for the prediction of DR diagnosis and compared the performance of XGB, DT, and RF models with that of the conventional logistic regression model. Among the models developed for predicting DR diagnosis using machine learning algorithms, the RF model showed the best performance.

Previous studies have developed deep learning systems that can detect and classify DR using fundus images. Dai et al. reported an average AUC of 0.955 for DR grading, while Bhimavarapu et al. achieved 99.41% accuracy using fundus images (15, 16). Fundus image-based DR prediction offers the advantage of high accuracy by directly capturing retinal pathology, enabling precise assessment of DR diagnosis and severity. However, in many regions, access to regular fundus imaging is limited due to various challenges (6–8). Additionally, AI-based fundus image analysis faces issues such as inconsistent image quality, poor pupil dilation, patient compliance, and suboptimal acquisition techniques, all of which require specialized equipment and trained personnel (17, 18). In such cases, identifying patients in need of DR screening using easily obtainable clinical data becomes clinically important. Our model leverages clinical information to facilitate early DR screening without requiring specialized equipment or personnel, making it more practical for routine clinical use. Furthermore, clinical variables are readily available and cost-effective. However, a limitation of our model is that its accuracy is lower compared to fundus imaging-based DR prediction. Additionally, the model’s performance may vary depending on the availability of the clinical variables included in the analysis.

There have also been several non-image-based machine learning studies for DR prediction. Islam et al. conducted a study using clinical information from a Chinese cohort and achieved an accuracy of 90.01% with an XGBoost-based model (19). Similarly, Zhao et al. validated a DR prediction model using baseline demographic and clinical characteristics in patients with type 2 diabetes mellitus, with the XGBoost model demonstrating the highest predictive performance (accuracy: 88.9%) (20). These two studies likely achieved higher accuracy due to their larger sample sizes compared to our study, because similar studies with relatively smaller sample sizes have reported results comparable to ours (accuracy, 73.5–79.5%) (21–23).

The potential integration of our optimized RF model into clinical decision support systems (CDSS) used by primary care physicians, such as those in internal medicine and family practice, offers several notable advantages. By utilizing relatively stable predictive outcomes, a CDSS can enhance diabetes management through more accurate and targeted referrals for ophthalmological evaluation. This could lead to significant reductions in healthcare costs by preventing unnecessary consultations and focusing resources on those most in need of specialized care (24, 25). Additionally, timely and accurate referrals can significantly improve patients’ quality of life by preventing vision loss and enabling better management of their condition (26, 27). This advantage could be maximized if this system is applied to large-scale populations, such as those in corporations, schools, and the military.

This study has several limitations. First, DR grading was not additionally assessed in all years, and only the presence or absence of DR was determined, making subanalysis based on DR grading not possible. Additionally, this model was confirmed an Asian ethnic group, requiring generalizability. To address these limitations, future research should include larger, more diverse patient groups and utilize detailed datasets to develop models that can more accurately identify individuals in need of hospital referrals for DR.

However, this study also has several strengths. First, it utilizes nationwide community-based data to ascertain the prevalence and risk factors of DR. This data provides a representative sample of the South Korean population. Second, the information was collected, examined, and interpreted using standardized protocols, ensuring objectivity and reliability of the data. Third, we developed an optimized model for DR risk assessment using various algorithms, enhancing the predictive accuracy and applicability of our findings. Our approach is convenient for the use in hospitals during routine health check-ups or diabetes management visits even without fundus examination. Furthermore, this method could effectively complement existing fundus photography-based models, providing a comprehensive and resource-efficient diagnostic tool for DR. As the next step, we have developed software to integrate this model into practical clinical use, and it is currently in the commercialization preparation stage.

## Conclusion

The prediction models for DR with machine learning techniques using nationwide survey data have the potential to be utilized as CDSS. Our results suggest that these models can aid in the management of DR by identifying patients at high risk, thereby facilitating timely ophthalmic referrals. Consequently, we believe this approach will be able to significantly contribute to the prevention of vision loss through improved DR risk management in people with diabetes.

## Data Availability

Publicly available datasets were analyzed in this study. This data can be found here: https://knhanes.kdca.go.kr.
